# Vascular endothelial growth factor (VEGF) expression is a prognostic factor for radiotherapy outcome in advanced carcinoma of the cervix

**DOI:** 10.1054/bjoc.2000.1319

**Published:** 2000-08-16

**Authors:** J A Loncaster, R A Cooper, J P Logue, S E Davidson, R D Hunter, C M L West

**Affiliations:** CRC Experimental Radiation Oncology Group, Paterson Institute for Cancer Research; Department of Clinical Oncology, Christie Hospital NHS Trust, Wilmslow Road, Manchester, M20 4BX, UK

**Keywords:** cervical carcinoma, vascular endothelial growth factor (VEGF), immunohistochemistry, prognosis

## Abstract

The aim of the study was to evaluate VEGF expression in tumour biopsies as a prognostic factor for radiotherapy outcome in advanced carcinoma of the cervix. A retrospective study was carried out on 100 patients. Pre-treatment tumour VEGF expression was examined immunohistochemically in formalin-fixed, paraffin-embedded biopsies using a widely available commercial antibody. A semi-quantitative analysis was made using a scoring system of 0, 1, 2, and 3, for increasing intensity of staining. High VEGF expression was associated with a poor prognosis. A univariate log rank analysis found a significant relationship with overall survival (*P* = 0.0008) and metastasis-free survival (*P* = 0.0062), but not local control (*P* = 0.23). There was no correlation between VEGF expression and disease stage, tumour differentiation, patient age, or tumour radiosensitivity (SF2). In a Cox multivariate analysis of survival VEGF expression was the most significant independent prognostic factor (*P* = 0.001). After allowing for VEGF only SF2 was a significant prognostic factor (*P* = 0.003). In conclusion, immunohistochemical analysis of VEGF expression is a highly significant and independent prognostic indicator of overall and metastasis-free survival for patients treated with radiotherapy for advanced carcinoma of the cervix. It is also a rapid and easy method that could be used in the clinical setting, to identify patients at high risk of failure with conventional radiotherapy who may benefit from novel approaches or chemoradiotherapy. © 2000 Cancer Research Campaign


*British Journal of Cancer* (2000) **83**(5), 620–625
© 2000 Cancer Research Campaign
doi: 10.1054/ bjoc.2000.1319, available online at http://www.idealibrary.com on IDEAL®

### Vascular endothelial growth factor (VEGF) expression is a prognostic factor for radiotherapy outcome in advanced carcinoma of the cervix

**JA Loncaster[1], RA Cooper[2], JP Logue[2], SE Davidson[2], RD Hunter[2] and CML West[1]**

[1]CRC Experimental Radiation Oncology Group, Paterson Institute for Cancer Research; and [2]Department of Clinical Oncology, Christie Hospital NHS Trust, Wilmslow Road, Manchester M20 4BX, UK

**Summary** The aim of the study was to evaluate VEGF expression in tumour biopsies as a prognostic factor for radiotherapy outcome in advanced carcinoma of the cervix. A retrospective study was carried out on 100 patients. Pre-treatment tumour VEGF expression was examined immunohistochemically in formalin-fixed, paraffin-embedded biopsies using a widely available commercial antibody. A semi-quantitative analysis was made using a scoring system of 0, 1, 2, and 3, for increasing intensity of staining. High VEGF expression was associated with a poor prognosis. A univariate log rank analysis found a significant relationship with overall survival ($P$ = 0.0008) and metastasis-free survival ($P$ = 0.0062), but not local control ($P$ = 0.23). There was no correlation between VEGF expression and disease stage, tumour differentiation, patient age, or tumour radiosensitivity (SF2). In a Cox multivariate analysis of survival VEGF expression was the most significant independent prognostic factor ($P$ = 0.001). After allowing for VEGF only SF2 was a significant prognostic factor ($P$ = 0.003). In conclusion, immunohistochemical analysis of VEGF expression is a highly significant and independent prognostic indicator of overall and metastasis-free survival for patients treated with radiotherapy for advanced carcinoma of the cervix. It is also a rapid and easy method that could be used in the clinical setting, to identify patients at high risk of failure with conventional radiotherapy who may benefit from novel approaches or chemoradiotherapy. © 2000 Cancer Research Campaign

**Keywords:** cervical carcinoma; vascular endothelial growth factor (VEGF); immunohistochemistry; prognosis

Angiogenesis is a necessary component of solid tumour growth (Folkman, 1990). It allows delivery of oxygen and nutrients to the tumour, and facilitates the transport of tumour cells to distant sites. In addition, secretion of certain cytokines and growth factors by the newly formed endothelial cells can have a paracrine effect on tumour growth by directly stimulating the tumour cells (Rak, 1996). The rate of angiogenesis is controlled by a number of factors secreted by the tumour cells. VEGF has been identified as one of the most important of these factors.

VEGF was initially detected as a factor, secreted by tumour cells into tissue culture medium or ascitic fluid, that caused normal blood vessels to become hyperpermeable (Senger et al, 1983). Subsequently it has been shown to be over-expressed in a number of human tumours, as well as in healing wounds, rheumatoid arthritis and delayed hypersensitivity reactions. It is a homodimeric heparin-binding glycoprotein that exists in 5 isoforms (*121*, *145*, *165*, *189* and *206*).

VEGF plays a critical role in tumour angiogenesis by increasing blood vessel permeability, and endothelial cell growth, proliferation, migration and differentiation (Ferrara, 1995). It may also facilitate extravasation of tumour cells and thereby the formation of metastases by degrading the tumour marginal extracellular matrix via activation of proteolytic enzymes (Pepper et al, 1991).

*Received 9 November 1999*
*Revised 27 April 2000*
*Accepted 8 May 2000*

*Correspondence to:* CML West

VEGF is one of a group of 'survival genes' whose expression is up-regulated in response to hypoxia via the transcription factor HIF-1 which exists as a functional stable dimer only in low oxygen tensions. In addition to HIF-1, a number of other cytokines can up-regulate VEGF, including known angiogenic growth factors such as bFGF, TGF$\alpha$ and epidermal growth factor. Mutant p53 and mutant ras have also been shown to up-regulate and enhance hypoxia-induced VEGF (Keiser et al, 1994; Rak et al, 1995) whereas wild type p53 has been shown to down-regulate VEGF transcription (Bouvet et al, 1998).

The receptors for VEGF are tyrosine kinases (VEGFR1 and VEGFR2) that are expressed predominantly on endothelial cells, but have also been identified on tumour cells (Boocock et al, 1995). This raises the possibility that VEGF may act not only as an angiogenic and vasopermeability factor, but also as a tumour cell autocrine factor.

High tumour expression of VEGF protein has been linked to poor outcome in a number of human tumour sites including stomach (Maeda et al, 1996), ovary (Paley et al, 1997; Yamamoto et al, 1997), oesophagus (Inoue et al, 1997; Uchida et al, 1998), breast (Linderholm et al, 1998), colorectum (Ishigami et al, 1998), lung (Fontanini et al, 1999) and bone (Lee et al, 1999). Although there is ample evidence that VEGF contributes to microinvasion in early stage disease (Guidi et al, 1995; Obermair et al, 1997), no large studies have examined the prognostic significance of VEGF expression in locally advanced carcinoma of the cervix. There is one small study that reported VEGF expression to have no predictive power in surgically treated disease (Hawighorst et al, 1998). As radiotherapy is the treatment of choice in locally advanced carcinoma of the cervix, we have examined the relationship between VEGF expression and radiotherapy outcome in cervical carcinoma, and assessed its independence as a prognostic factor using multivariate analysis.

### MATERIALS AND METHODS

### Patients

A total of 100 patients with locally advanced (i.e., bulky stage Ib to IIIb) carcinoma of the cervix were included in the study. All the patients gave prior written consent to allow tumour biopsies to be taken for research purposes at the time of their examinatiosn under anaesthesia (EUA). An EUA was undertaken on all patients in order to establish staging according to FIGO criteria. Of the 100 patients, 94 had squamous cell carcinoma, 5 had adenocarcinoma and one had adenosquamous carcinoma. All patients were treated with radiotherapy with curative intent according to the standard techniques of the Manchester school (West et al, 1993). Patients were reviewed in specialist oncology clinics, following a standard protocol, for a minimum of five years. Further follow up information was obtained from questionnaires to general practitioners. The median follow-up time was 49 months (range 3–117 months). For surviving patients, the median follow-up was 80 months (range 28–117 months). The sites of any disease relapse were identified clinically and radiologically, and where appropriate was confirmed on biopsy. The recurrences were then classified as being either local (i.e. within the radiotherapy field) or metastatic (i.e. outside the radiotherapy field). Pelvic side-wall recurrence was taken as local for tumours that had received external beam radiotherapy or as metastatic for tumours treated by intracavity radiotherapy alone. Data were also available for tumour radiosensitivity, measured as the surviving fraction at 2 Gy (SF2) on 62 patients from previous work by the group (West et al, 1993).

### Assessment of tumour VEGF protein expression

Measurements were made on pre-treatment biopsy material. Formalin-fixed, paraffin-embedded, 4-µm sections were prepared from the biopsy specimens, and were immunostained using a Dako envision™+ system. Following deparaffination and rehydration, antigen retrieval was performed by microwaving for 25 minutes in Tris-HCl 0.05M, pH 8.5, containing EDTA 0.001M. After resting for 15 minutes an endogenous peroxidase block, supplied with the kit, was applied for 5 minutes. Following washing, the samples were incubated with 10% casein (Vector) in TBS (blocking buffer) for 10 minutes. Sections were then incubated for 30 minutes at room temperature with anti-VEGF polyclonal antibody (Santa Cruz Biotechnology Inc., Santa Cruz, CA) at 1 in 200 dilution. The antibody was chosen for a number of reasons. First, it is widely available and used in clinical studies (e.g., Kuhnen et al, 2000; Parliament et al, 2000). Second, it was used in several studies and shown to have prognostic significance (Maeda et al, 1996; Yamamoto et al, 1997). Third, others have confirmed the reliability of the antibody and shown it to be highly specific in immunohistochemical reactions of human tumours (Ohta et al, 1996; Takahashi et al, 1996; Itakura et al, 1997).

A solution of normal rabbit immunoglobulin was diluted to the same concentration and substituted for the primary antibody to act as a negative control. Following washing, the sections were incubated at room temperature for 30 minutes with a peroxidase labelled polymer conjugated to goat-anti-rabbit immunoglobulins in Tris-HCl buffer (supplied in the kit). A substrate-chromogen solution containing 3,3′-diaminobenzidine in a buffered substrate solution (pH 7.5) containing hydrogen peroxide, again supplied with the kit, was applied for 5 minutes. After rinsing in water, the slides were lightly counter-stained with Gills haematoxylin, dehydrated and mounted.

The VEGF expression in the tumour cells was evaluated using a semi-quantitative scoring system: 0 for absence of immunostaining, 1 for light staining, 2 for moderate staining, and 3 for heavy staining. Any staining of the tumour stroma was ignored in this assessment. Batch to batch variation was excluded by running sections from the same biopsy through more than one batch, and running one biopsy section through all the batches.

### Statistical analysis

Survival was analysed using the Kaplan-Meier method, and prognostic factors were assessed by log-rank analysis. Univariate and bivariate analyses were made of overall survival, metastasis-free survival, and local recurrence-free survival. Patients were stratified by their VEGF score as well as other putative prognostic factors (age, stage, tumour differentiation and SF2). A stepwise multivariate Cox regression analysis was also performed to further test the independence of VEGF from other parameters. The distribution of the VEGF score in relation to the tumour and patient characteristics was investigated using Fisher's exact test. Correlations between variables were obtained using Spearman's rank correlation.

### RESULTS

### Scoring reproducibility

Intra-observer variation for scoring of VEGF intensity was tested on a random sample of 33 slides, scored by the same observer (JAL) at least one month after the initial scoring. The results correlated significantly ($r = 0.80$, $P < 0.001$). Where the score differed from the initial score, it did so only by one scale point. Inter-observer variability was assessed by a different observer (CMLW) re-scoring 20 of the slides. There was a significant correlation between the two observers' scores ($r = 0.93$, $P < 0.001$). Again, when any differences in scores were identified, they varied by only one scale point.

Batch to batch variation was assessed by scoring sections from the same biopsies that had been run through more than one batch. Out of fifteen repeat sections, the score varied by one scale point in one section only. The other 14 sections were scored identically, independent of batch.

### VEGF expression

Immunostaining was essentially cytoplasmic but occasionally seen in the nuclei. It was located primarily in the tumour cells, although weaker staining was seen in the stroma of most sections. There was no obvious spatial relationship to the tumour blood vessels. In most cases where positive staining was identified, the intensity remained fairly homogeneous thoughout the tumour section.

© 2000 Cancer Research Campaign 

**Table 1** Summary of the distribution of patients according to VEGF intensity

| Parameter | | *n* | VEGF 0 | VEGF 1 | VEGF 2 | VEGF 3 | *P*[a] |
|---|---|---|---|---|---|---|---|
| Stage | I | 35 | 1 | 12 | 10 | 12 | |
| | II | 36 | 4 | 8 | 14 | 10 | |
| | III | 29 | 1 | 7 | 12 | 9 | 0.52 |
| Age | < 49 years | 53 | 4 | 17 | 18 | 14 | |
| | > 49 years | 47 | 2 | 10 | 18 | 17 | 0.61 |
| Histology | SCC[b] | 94 | 4 | 25 | 35 | 30 | |
| | Adenocarcinoma | 5 | 2 | 2 | 0 | 1 | |
| | Adenosquamous | 1 | 0 | 0 | 1 | 0 | 0.10 |
| Differentiation | Well | 16 | 2 | 7 | 4 | 3 | |
| | Moderate | 62 | 4 | 14 | 24 | 20 | |
| | Poor | 22 | 0 | 6 | 8 | 8 | 0.29 |
| Radiosensitivity | SF2 < median | 35 | 3 | 8 | 15 | 9 | |
| | SF2 > median | 27 | 0 | 5 | 9 | 13 | 0.24 |

[a]Fisher's exact test for differences in the distribution of patients according to the parameters listed; [b]squamous cell carcinoma

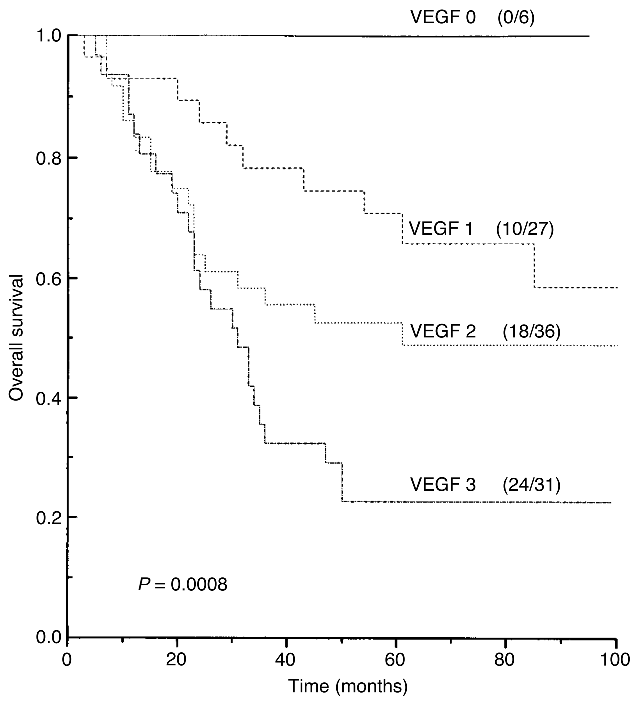

**Figure 1** Overall survival in relation to VEGF expression for 100 cervical cancer patients treated with radiotherapy with a median follow-up for surviving patients of 80 months. Patients were stratified according to the intensity of VEGF expression. The numbers of disease-related deaths and patients in each arm are indicated

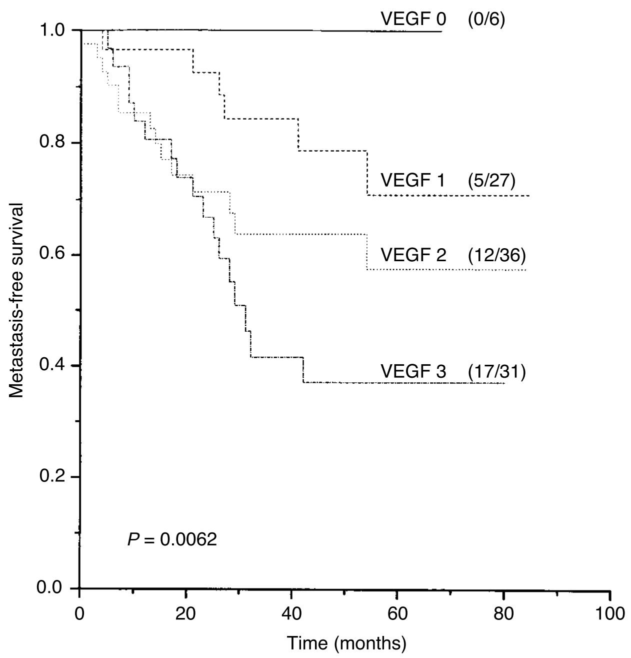

**Figure 2** Metastasis-free survival in relation to VEGF expression for 100 cervical cancer patients treated with radiotherapy with a median follow-up for surviving patients of 80 months. Patients were stratified according to the intensity of VEGF expression. The numbers of events and patients in each arm are indicated

Tumour positivity for VEGF expression was identified in 94% of the sections.

### Distribution of patients according to VEGF intensity

Table 1 summarises the distribution of the 100 patients according to tumour VEGF intensity and various clinical and biological characteristics. Using Fisher's exact test no significant differences were seen in the distribution of patients according to VEGF expression and any of the characteristics studied. Using Spearman's rank analysis there was also no significant correlation between VEGF score and patient age (r = 0.12, $P = 0.23$), disease stage (r = 0.57, $P = 0.55$) or SF2 (r = 0.15, $P = 0.24$).

### Correlation with outcome

Tumour VEGF expression was a highly significant prognostic factor for survival (Figure 1) and metastasis-free survival (Figure 2), but not for local control (Figure 3). In univariate log-rank analysis only VEGF expression and radiosensitivity were predictive of treatment outcome (Table 2). Bivariate log-rank analyses were carried out in order to test the independence of VEGF expression from other potential prognostic factors. After allowing for patient age, tumour stage, differentiation, and SF2, the level of VEGF expression remained a significant prognostic factor for both overall and metastasis-free survival (Table 3).

 © 2000 Cancer Research Campaign

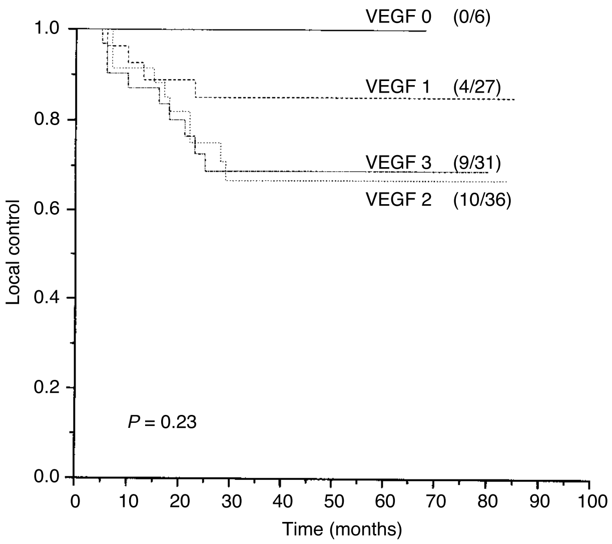

**Figure 3** Local control in relation to VEGF expression for 100 cervical cancer patients treated with radiotherapy with a median follow-up for surviving patients of 80 months. Patients were stratified according to the intensity of VEGF expression. The numbers of events and patients in each arm are indicated

A Cox multiple regression analysis was performed to further evaluate the independence of VEGF expression as a prognostic factor (Table 4). The level of VEGF expression emerged as the most important prognostic indicator for overall survival. After allowing for VEGF intensity only SF2 was an independent prognostic factor for overall survival. VEGF expression and SF2 were also significant independent predictors for metastasis-free survival. Only SF2 was an independent prognostic factor for local recurrence.

### DISCUSSION

### VEGF as a prognostic factor

This study illustrates the importance of tumour VEGF expression as a significant and independent prognostic factor in cervical carcinoma. The work supports the findings of other groups looking at different tumour sites and involving treatment predominantly by surgery (Maeda et al, 1996; Paley et al, 1997; Yamamoto et al, 1997; Inoue et al, 1997; Ishigami et al, 1998; Linderholm et al, 1998; Uchida et al, 1998; Fontanini et al, 1999; Lee et al, 1999). VEGF expression was significantly associated with both overall and metastasis-free survival but not local control. This suggests that VEGF expression is primarily reflecting the metastatic potential of a tumour rather than response to radiotherapy. The latter suggestion is supported by the known roles of VEGF in promoting metastatic spread by increasing blood vessel permeability and establishing a new blood supply required for tumour growth (Ferrara, 1995). It is also supported by the proposed role of VEGF in the induction of the plasminogen activation system (Pepper et al, 1991), which would increase the metastatic potential of the tumour via degradation of the marginal extracellular matrix.

Previous work by us demonstrated intrinsic radiosensitivity (SF2) to be a strong predictor of outcome following radiotherapy for cervical carcinoma (West et al, 1993). This study has shown that SF2 remains the strongest predictor of local control but that VEGF expression is the strongest predictor of overall survival and is entirely independent of SF2.

We have shown that the method of assessing VEGF expression is highly reproducible. There is minimal inter-batch variation and good correlation between inter- and intra-observer scores. The method is rapid and results can be available within days of taking the biopsy. It could be easily incorporated into routine clinical practice, at a time when treatment decisions are being made.

### Potential applications for assessing tumour VEGF expression

Patients with a high intensity of tumour VEGF expression have been shown to have a particularly poor prognosis. This information may be used in future to individualize treatment. There are a number of potential approaches. First, patients with high VEGF expression could be offered concurrent chemotherapy. Patients with locally advanced cervical carcinomas presently receive

**Table 2** Univariate log-rank analysis of putative prognostic factors for outcome following radical radiotherapy for carcinoma of the cervix. The *P* values for each factor are given

| Parameter | *n* | Overall survival | Metastasis-free survival | Local control |
|---|---|---|---|---|
| Stage | 100 | 0.17 | 0.29 | 0.20 |
| Differentiation | 100 | 0.27 | 0.51 | 0.92 |
| Age | 100 | 0.85 | 0.19 | 0.71 |
| SF2[a] | 62 | < 0.001 | 0.002 | 0.003 |
| VEGF intensity | 100 | 0.0008 | 0.0062 | 0.23 |

[a]Surviving fraction at 2 Gy

**Table 3** Bivariate stratified log-rank analyses showing the significance of VEGF expression as a prognostic factor for cervical carcinoma after allowing for the listed parameters

| Parameter | *n* | Overall survival *P* | Metastasis-free survival *P* | Local control *P* |
|---|---|---|---|---|
| Stage | 100 | 0.004 | 0.020 | 0.30 |
| Differentiation | 100 | 0.005 | 0.055 | 0.36 |
| Age | 100 | 0.005 | 0.017 | 0.26 |
| SF2[a] | 62 | 0.005 | 0.023 | 0.24 |

[a]Surviving fraction at 2 Gy

**Table 4** Cox multiple regression analysis of significant prognostic factors for 62 patients[a]

| | Overall survival | Metastasis-free survival | Local control | | | |
|---|---|---|---|---|---|---|
| | ***P***[b] | **RR**[c] | ***P*** | **RR** | ***P*** | **RR** |
| SF2[d] | 0.003 | 3.2 | 0.018 | 3.3 | 0.008 | 1.45 |
| VEGF | 0.001 | 2.3 | 0.021 | 2.2 | ns[e] | |

[a]Analysis includes stage, pathology, VEGF, age, SF2 and grade; [b]significance of the parameter as a prognostic factor; [c]relative risk; [d]surviving fraction at 2 Gy; [e]not significant radical radiotherapy as the standard treatment. However in the light of recent reports of improved survival for patients treated with concurrent cisplatin-containing chemoradiotherapy (Keys et al, 1999; Morris et al, 1999; Peters et al, 1999; Rose et al, 1999; Whitney et al, 1999) many clinicians have been encouraged to adopt this as their standard approach. As yet there is little information on the late toxicity following this combined treatment. The patients in this study with low tumour expression of VEGF have been shown to have a good overall survival rate, and therefore may be less likely to benefit from concurrent chemotherapy and its potential additional toxicities. VEGF expression could be used to guide the use of concurrent chemotherapy and allow the selection of patients with good prognosis tumours who could be treated with radiotherapy alone. Second, it has been shown that prolonged exposure to hypoxia can induce VEGF mRNA in cervical cell lines (Chiarotto and Hill, 1999), although this relationship has not been demonstrated in vivo in solid tumours (Raleigh et al, 1998). If VEGF production is directly linked to hypoxia in solid tumours then it is possible that hypoxic cell modification may reduce VEGF expression. Third, analysis of VEGF expression could indicate the patients most likely to benefit from therapy targeting VEGF-mediated tumour angiogenesis. Anti-VEGF antibodies have the ability to inhibit the growth of several tumour cell lines in nude mice (Kim et al, 1993; Warren et al, 1995) and can inhibit ascites formation in mice transplanted with ascites-producing tumours (Nagy et al, 1995). The safety and efficacy of this strategy is presently being tested in humans (Presta et al, 1997). Fourth, the use of retroviruses encoding dominant-negative mutant VEGF receptors can block the effects of VEGF and retard tumour growth (Millaeur et al, 1994). Finally, gene therapy involving the replacement of mutant p53 by restoration of wt p53 can down-regulate VEGF expression and thereby inhibit angiogenesis (Bouvet et al, 1998).

In conclusion, tumour expression of VEGF as assessed by immunohistochemical analysis is a highly significant and independent prognostic factor for patients with carcinoma of the cervix treated with radiotherapy. The method used is quick, simple and reproducible, and could be used to aid treatment stratification and direct the use of anti-angiogenic therapies.

### ACKNOWLEDGEMENT

The authors would like to thank Dr Stephen Roberts for his assistance with the statistical analysis.

### REFERENCES

Boocock CA, Charnock-Jones DS, Sharkey AM, McLaren J, Barker PJ, Wright KA, Twentyman PR and Smith SK (1995) Expression of vascular endothelial growth factor and its receptors flt and KDR in ovarian carcinoma. *J Natl Cancer Institute* **87**: 506–516

Bouvet M, Ellis LM, Nishizaki M, Fujiwara T, Liu W, Bucana CD, Fang B, Lee J and Roth JA (1998) Adenovirus-mediated wild-type p53 gene transfer down-regulates vascular endothelial growth factor expression and inhibits angiogenesis in human colon cancer. *Cancer Res* **58**: 2288–2292

Chiarotto JA and Hill RP (1999) A quantitative analysis of the reduction in oxygen levels required to induce up-regulation of vascular endothelial growth factor (VEGF) mRNA in cervical cell lines. *Br J Cancer* **80**: 1518–1524

Ferrara N (1995) The role of vascular endothelial growth factor in pathological angiogenesis. *Breast Cancer Res Treat* **36**: 127–137

Folkman J (1990) What is the evidence that tumors are angiogenesis dependent? *J Natl Cancer Institute* **82**: 4–6

Fontanini G, Boldrini L, Chine S, Pisaturo F, Basalo F, Calcinai A, Lucchi M, Angeletti CA and Bevilacqua G (1999) Expression of vascular endothelial growth factor mRNA in non-small cell lung carcinomas. *Br J Cancer* **79**: 363–369

Guidi A, Abu-Jawdeh G, Berse B, Jackman RW, Tognazzi K, Dvorak HF and Brown LF (1995) Vascular permeability factor (vascular endothelial growth factor) expression and angiogenesis in cervical neoplasia. *J Natl Cancer Institute* **87**: 1237–1245

Hawighorst H, Weikel W, Knapstein PG, Knopp MV, Zuna I, Schonberg SO, Vaupel P and van Kaick G (1998) Angiogenic activity of cervical carcinoma: Assessment by functional magnetic resonance imaging-based parameters and a histomorphological approach in correlation with disease outcome *Clin Cancer Res* **4**: 2305–2312

Inoue K, Ozeki Y, Suganuma T, Sugiura T and Tanaka S (1997) Vascular endothelial growth factor expression in primary esophageal squamous cell carcinoma. *Cancer* **79**: 206–213

Ishigami SI, Arii S, Furutani M, Niwano M, Harada T, Mizumoto M, Mori A and Imamura M (1998) Predictive value of vascular endothelial growth factor (VEGF) in metastasis and prognosis of human colorectal cancer. *Br J Cancer* **78**: 1379–1384

Itakura J, Ishiwata T, Freiss H, Fujii H, Matsumoto Y, Buchler MW and Korc M (1997) Enhanced expression of vascular endothelial growth factor in human pancreatic cancer correlates with local disease progression. *Clin Cancer Res* **3**: 1309–1316

Keiser A, Weich HA, Brandner G, Marme D and Kolch W (1994) Mutant p53 potentiates protein kinase C induction of vascular endothelial growth factor expression. *Oncogene* **9**: 963–969

Keys HM, Bundy BN and Stehman FB (1999) Cisplatin, radiation, and adjuvant hysterectomy for bulky stage IB cervical carcinoma. *N Engl J Med* **340**: 1154–1161

Kim KJ, Li B, Winer J, Armanini M, Gillett N, Phillips HS and Ferrara N (1993) Inhibition of vascular endothelial growth factor-induced angiogenesis suppresses tumour growth *in vivo*. *Nature* **362**: 576–579

Kuhnen C, Lehnhardt M, Tolnay E, Muehlberger T, Vogt PM and Muller K-M (2000) Patterns of expression and secretion of vascular endothelial growth factor in malignant soft-tissue tumours. *J Cancer Res Clin Oncol* **126**: 219–225

Lee YH, Tokunaga T, Oshika Y, Suto R, Yanagisawa K, Tomisawa M, Fukuda H, Abe S, Tateishi A, Kijima H, Yamazaki H, Tamaoki N, Ueyama Y and Nakamura M (1999) Cell-retained isoforms of vascular endothelial growth factor (VEGF) are correlated with poor prognosis in osteosarcoma. *Eur J cancer* **35**: 1089–1093

Linderholm B, Tavelin B, Grankvist K and Henriksson R (1998) Vascular endothelial growth factor is of high prognostic value in node-negative breast carcinoma. *J Clin Onc* **16**: 3121–3128

Maeda K, Chung YS, Ogawa Y, Takatsuka S, Kang S, Ogawa M, Sawada T and Sowa M (1996) Prognostic value of vascular endothelial growth factor expression in gastric carcinoma. *Cancer* **77**: 858–863

Millaeur B, Shawver KL and Plate KH (1994) Glioblastoma growth inhibited in vivo by a dominant-negative Flk-1 mutant. *Nature* **367**: 576–579

Morris M, Eifel PJ and Lu J (1999) Pelvic radiation with concurrent chemotherapy compared with pelvic and paraaortic radiation for high risk cervical cancer. *N Engl J Med* **340**: 1137–1143

 *© 2000 Cancer Research Campaign*

Nagy JA, Masse EM, Herzberg KT, Myers MS, Yeo K, Sioussat T and Dvorak H (1995) Pathogenesis of ascites tumour growth: vascular permeability factor, vascular hyperpermeability, and ascites fluid formation. *Cancer Res* **55**: 360–368

Obermair A, Bancher-Todesca D, Bilgi S, Kaider A, Kohlberger P, Mullauer-Ertl S, Leodolter S and Gitsch G (1997) Correlation of vascular endothelial growth factor expression and microvessel density in cervical intraepithelial neoplasia. *J Natl Cancer Inst* **89**: 1212–1217

Ohta Y, Endo Y, Tanaka M, Shimizu J, Oda M, Hayashi Y, Watanabe Y and Sasaki T (1996) Significance of vascular endothelial growth factor messnger RNA expression in primary lung cancer. *Clin Cancer Res* **2**: 1411–1416

Paley PJ, Staskus KA, Gebhard K, Mohanraj D, Twiggs LB and Ramakrishnan S (1997) Vascular endothelial growth factor expression in early stage ovarian carcinoma. *Cancer* **80**: 98–106

Parliament MB, Allalunis-Turner MJ, Franko AJ, Olive PL, Mandyam R, Santos C and Wolokoff B (2000) Vascular endothelial growth factor expression is independent of hypoxia in human malignant glioma spheroids and tumours. *Br J Cancer* **82**: 635–641

Peters WA III, Liu PY and Barrett R (1999) Cisplatin, 5-fluorouracil plus radiation therapy are superior to radiation therapy as adjunctive therapy in high-risk, early stage carcinoma of the cervix after radical hysterectomy and pelvic lymphadenectomy. *Proc Soc Gynecol Oncol* **28** (abstr)

Pepper MS, Ferrara N and Orchi L (1991) Vascular endothelial growth factor (VEGF) induces plasminogen activators and plasminogen activator inhibitor-1 in microvascular endothelial cells. *Biochem Biophys Res Commun* **181**: 902–906

Presta LG, Chen H, O'Conner SJ, Chisholm V, Winkler M and Ferrara N (1997) Humanisation of an anti-vascular endothelial growth factor monoclonal antibody for the therapy of solid tumours and other disorders. *Cancer Res* **57**: 4593–4599

Rak J (1996) Reciprocal paracrine interactions between tumour cells and endothelial cells: the 'angiogenesis progression' hypothesis. *Eur J Cancer* **32A**: 2438–2450

Rak J, Mitsuhashi Y and Bayko L (1995) Mutant ras oncogenes up-regulate VEGF/VPF expression-implications for induction and inhibition of tumour angiogenesis. *Cancer Res* **55**: 4574–4580

Raleigh JA, Calkins-Adams DP, Rinker LH, Ballenger CA, Weisser MC, Fowler WC, Novotny D and Varia M (1998) Hypoxia and vascular endothelial growth factor expression in human squamous cell carcinomas using pimonidazole as a hypoxia marker. *Cancer Res* **58**: 3765–3768

Senger DR, Galli SJ, Peruzzi CA, Harvey VS and Dvorak HF (1983) Tumour cells secrete a vascular permeability factor that promotes accumulation of ascites fluid. *Science* **219**: 983–985

Takahashi Y, Cleary KR, Mai M, Kitadai Y, Bucana CD and Ellis LM (1996) Significance of vessel count and vascular endothelial growth factor and its receptor (KDR) in intestinal-type gastric cancer. *Clin Cancer Res* **2**: 1679–1684

Uchida S, Shimada Y, Watanabe G, Tanaka H, Shobagaki I, Miyahara T, Ishigami S, Arii S and Imamura M (1998) In oesophageal squamous cell carcinoma vascular endothelial growth factor is associated with p53 mutation, advanced stage and poor prognosis. *Br J Cancer* **77**: 1704–1709

Warren RA, Yuan H and Matli MR (1995) Regulation by vascular endothelial growth factor of human colon cancer tumorigenesis in a mouse model of experimental liver metastases. *J Clin Invest* **95**: 1789–1797

West CML, Davidson SE, Roberts SA and Hunter RD (1993) Intrinsic radiosensitivity and prediction of patient response to radiotherapy for carcinoma of the cervix. *Br J Cancer* **68**: 819–823

Whitney CW, Sause W, Bundy BN, Malfetano JH, Hannigan EV, Fowler WC, Clarke-Pearson DL and Liao S (1999) Randomised comparison of fluorouracil plus cisplatin versus hydroxyurea as an adjunct to radiation therapy in stage IIB-IVA carcinoma of the cervix with negative paraaortic lymph nodes. *J Clin Oncol* **17**: 1339–1343

Yamamoto S, Konishi I, Mandai M, Kuroda H, Komatsu T, Nanbu K, Sakahara H and Mori T (1997) Expression of vascular endothelial growth factor (VEGF) in epithelial ovarian neoplasms: correlation with clinicopathology and patient survival, and analysis of serum VEGF levels. *Br J Cancer* **76**: 1221–1227

*© 2000 Cancer Research Campaign*
